# Genetic Inactivation of Two-Pore Channel 1 Impairs Spatial Learning and Memory

**DOI:** 10.1007/s10519-020-10011-1

**Published:** 2020-09-05

**Authors:** Robert Theodor Mallmann, Norbert Klugbauer

**Affiliations:** 1 Institut für Experimentelle und Klinische Pharmakologie und Toxikologie, Medizinische Fakultät, Albert-Ludwigs-Universität, Freiburg, Germany; 2 Institut für Experimentelle und Klinische Pharmakologie und Toxikologie, Medizinische Fakultät, Universität Freiburg, Albertstr. 25, 79104 Freiburg, Germany

**Keywords:** Two-pore channel, TPC1, NAADP, Morris water maze, Spatial learning

## Abstract

Two-pore channels (TPCs) constitute a small family of cation channels that are localized in membranes of endosomal and lysosomal compartments. Although their roles for vesicular fusion and endolysosomal trafficking have been investigated, our knowledge on their expression pattern and higher order functions in the murine brain is still limited. Western blot analysis indicated a broad expression of TPC1 in the neocortex, cerebellum and hippocampus. In order to investigate the consequences of the genetic inactivation of TPC1, we performed a set of behavioural studies with TPC1^−/−^ mice. TPC1^−/−^ mice were analysed for an altered motor coordination and grip-strength, exploratory drive and anxiety as well as learning and memory. TPC1^−/−^ mice did not show any differences in their exploratory drive or in their anxiety levels. There were also no differences in spontaneous activity or motor performance. However, the Morris water maze test uncovered a deficit in spatial learning and memory in TPC1^−/−^ mice.

**Electronic supplementary material:**

The online version of this article (10.1007/s10519-020-10011-1) contains supplementary material, which is available to authorized users.

## Introduction

Two-pore channels (TPCs) constitute a small family of intracellularly localized cation channels. In mice and humans, only two TPCs have been identified, though, other species express three functional *tpc*-genes. Evolutionarily TPCs form intermediates between one-domain TRP and four-domain voltage gated Na^+^ or Ca^2+^ channels. TPCs are found in membranes of small and acidic intracellular organelles such as endosomes and lysosomes. Within these compartments, they show a smooth transition from TPC1 predominantly found in early and recycling endosomes to TPC2 mainly expressed in late endosomes and lysosomes (reviewed in Galione et al. 2009, 2010; Patel 2015). Diversity of TPCs is even wider since the TPC1 gene is expressed not only as full-length isoform TPC1A, but also as an N-terminal truncated variant TPC1B (Ruas et al. 2014). Both isoforms demonstrate different co-localizations with markers of early and recycling endosomes and it is highly probable that the isoforms contribute to different endolysosomal functions. TPCs are assumed to be involved in the regulation of endocytic and recycling/degradation processes, such as vesicle trafficking, sorting and membrane fusion events (Grimm et al. 2012, 2014).

The importance of TPC1 and TPC2 for intracellular vesicle trafficking has also been shown in pathophysiological contexts such as for processing of bacterial protein toxins and for uptake of certain viruses. For instance, the entry of Ebola viruses depends on functional TPCs so that pharmacological blockade or genetic inactivation of TPCs causes a trapping of virus particles in lysosomal compartments (Sakurai et al. 2015). The importance of TPC1 for the internalization and processing of bacterial protein toxins was systematically elaborated for the main intracellular uptake routes, which are typically hijacked by these toxins (Castonguay et al. 2017). Deletion of TPC1 interferes with uptake of toxins and delayes intoxication.

These examples illustrate that our knowledge on the intracellular role of TPCs is currently increasing and allows novel insights into endolysosomal trafficking processes. However, a solid understanding on higher order functions of TPCs is still limited and is currently concentrated on TPC2. So far, there are several reports highlighting the role of TPC2 in liver, melanocytes, heart and a Parkinson´s disease model (Ambrosio et al. 2016; Chao et al. 2017; Galione 2015; Grimm et al. 2014; Hockey et al. 2015). In contrast to this work, very few knockout-based studies investigated physiological roles of TPC1. For instance, Arndt and colleagues showed that TPC1 is expressed in the acrosomal region of mammalian spermatozoa (Arndt et al. 2014). There, TPC1 shows a high degree of co-localization with NAADP-binding sites. NAADP is a main ligand of TPCs and activates TPCs presumably via an accessory, but so far not identified subunit. Genetic inactivation of TPC1 inhibits NAADP-induced acrosomal exocytosis in the concentration-range of about 1 µM NAADP.

Thus, our current knowledge on higher order functions of TPC1 is rather limited and very selective. A systematic review on the consequences of a genetic inactivation of TPC1 in mice for complex behavior is still missing. In order to assess possible roles of TPC1 in the brain, earlier work focusing on NAADP-mediated effects should be taken into account. NAADP-signalling has been described in various species and is documented in numerous publications. For instance, NAADP was shown to participate in neurotransmitter release, in the modulation of neuronal excitability, in neurite outgrowth and neuronal differentiation and finally, NAADP-signalling was linked to activation of neurotransmitter receptors (Brailoiu et al. 2001, 2006, 2009; Chameau et al. 2001; Foster et al. 2018; Padamsey et al. 2017; Zhang et al. 2013). The enhancement of transmitter release by NAADP was demonstrated at the frog neuromuscular junction and at Aplysia cholinergic neurons (Brailoiu et al. 2001; Chameau et al. 2001). Taken together, these data indicate that NAADP-signalling is well described for neurotransmission and neuronal development. A TPC1 knockout model should allow to translate these observations into the context of higher order functions, i.e. in behavioral studies.

## Materials and methods

### Generation of TPC1 knock out mice and housing of animals

**Fig. 1:**
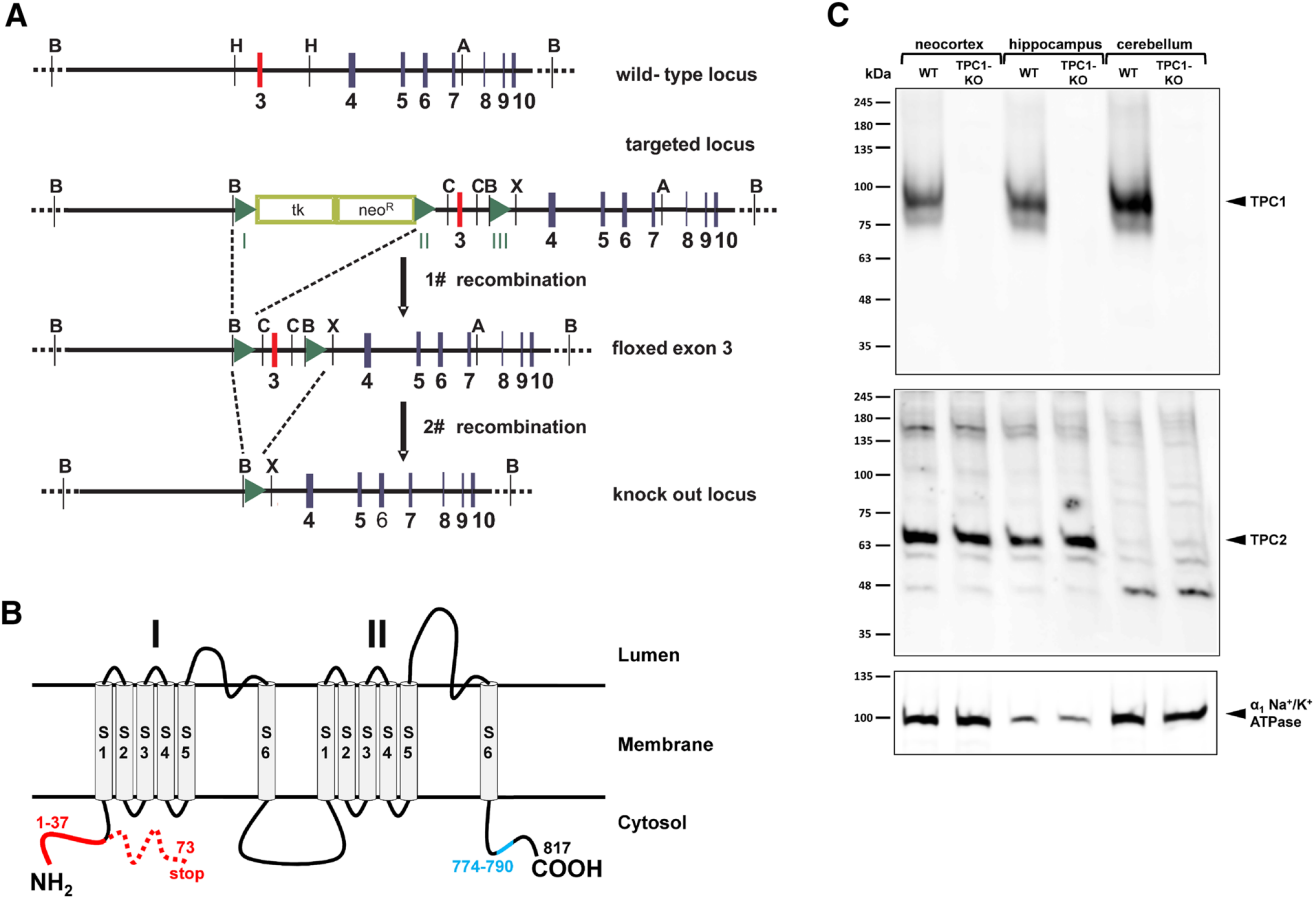
Strategy for the genetic inactivation of TPC1 and western blot analysis of murine brain regions. **a** Schematic representation of the wild type, targeted, “floxed” exon 3 and knock out *tpc1* gene locus. The numbers indicate the exon number. The TPC1 knock out model was generated by two rounds of recombination. First, the neomycin resistance cassette was removed by Cre-mediated recombination in targeted ES cells. Second, the knock out locus was generated by crossing mice containing a floxed exon 3 allele with ROSA26 Cre mice. *A* Acc65I, *B* BamHI, *C* ClaI, *H* HindIII, *X* XhoI. **b** Schematic representation of the TPC1 transmembrane topology and consequences of deletion of exon 3. Cre-mediated deletion of exon 3 causes a frameshift and a premature stop codon resulting in a 73 amino acid residues N-terminal fragment. The amino acid sequence (aa 774–790) for generating the TPC1 antibody is indicated in blue. **c** Representative western blot of membrane preparations obtained from WT and TPC1 knock out mouse brains. First line: Expression of TPC1 in neocortex, hippocampus and cerebellum of WT and TPC1 knock out mice. Second line: Corresponding brain regions do not show a compensatory upregulation of TPC2 in TPC1 knock out mice. Third line: Loading control using an antibody for Na^+^/K^+^-ATPase (Color figure online)

The strategy for generating a global TPC1 knock out line is shown in Fig. 1a. In short, we generated first a mouse line containing a “floxed” exon 3 TPC1 allele. The targeting vector used encompasses a “floxed” neo-tk cassette upstream of exon 3 and a single loxP site downstream of exon 3. The cloning strategy was based on the restriction sites depicted in Fig. 1a. After homologous recombination of the targeting vector into embryonic stem (ES) cells, correct gene insertion was verified by Southern blotting and by sequence analysis of genomic PCR fragments. ES cells containing the targeted locus were transfected with a plasmid encoding the Cre-recombinase and selected for deletion of the “floxed” neo-tk cassette. ES cells containing only the “floxed” exon 3 allele were used to establish the transgenic mouse line. These animals were crossed with ROSA26 Cre mice to obtain a global TPC1 knock-out line. Deletion of exon 3 (128 bp) leads to a frame-shift in the TPC1 gene and to a premature stop codon (Fig. 1b). Genetic manipulations were verified by sequence analysis and deletion of TPC1 by western blot analysis (Fig. 1c).

All animal experiments were performed in compliance with the German animal protection law (TierSchG). Mice were housed and handled in accordance with good animal practice as defined by FELASA (www.felasa.eu/guidelines.php) and the national animal welfare body GV-SOLAS (www.gv-solas.de/index.html). The animal welfare committee of the University of Freiburg as well as local authorities (Regierungspräsidium Freiburg) approved all animal experiments. Animals were housed in a temperature and humidity controlled vivarium with a 12 h light-dark cycle, food and water were available ad libitum.

### Behavioral studies

Behavioral phenotyping was performed with adult male mice only (genetic background: C57/Bl6 N), the age of mice is specified below for each study. Only age-matched littermate mice were used for the tests. To exclude possible influences of complex environmental enrichment on behavior, only nest-building material was available to the animals (van Praag et al. 2000). Behavioral phenotyping experiments were performed during the second half of day cycle. Prior to behavioural testing, animals were transferred in their home cages to the phenotyping lab and allowed to acclimate for at least 1 h. Activity and behaviour of mice were observed using an automated video tracking system for recording and analysis (VideoMot2 system V6.01, TSE, Bad Homburg, Germany). The experimenter was blind to the genotype of all animals tested. Statistical data of behavioral studies were analysed using SigmaPlot 13.0 (Erkrath, Germany). P < 0.05 was considered as significant. The number of mice was calculated according to the statistical needs of our standardised behavioural studies. Those are described in Crawley (2007) and Jones and Mormède (1999).

Dimensions and material of devices and detailed conditions of behavioral studies were performed as described in Mallmann et al. (2013). In short:

- Elevated plus maze: This test was performed with 12 to 21 weeks old mice. Elevated plus maze device consisted of two open and two closed arms each of 30 × 5 cm, closed arms were surrounded by a 15 cm high wall. All arms emerged from a central platform which was elevated 45 cm above the floor. Entry and duration of the mice in each arm was continuously assessed during 9 min.
- Free running wheel activity: Single caged, adult mice (12–28 weeks old) were allowed for voluntary wheel running during 21 days. After 14 days, standard wheels (regular rung configuration) were replaced by “complex wheels”. Complex wheels were prepared by removing particular rungs to create an irregular rung configuration (identical to that described in Liebetanz and Merkler 2006). Wheel running activity was recorded and average running speed and distances was calculated.
- Open field: This test was performed with 10 weeks old mice. The open field consisted of a square of 50 × 50 cm surrounded by a 35 cm wall. The behavior in the open field was recorded for 20 min. Evaluation of data sets included time spent in the central area, covered distance at central area, average distance to “open field” center and total covered distance.
- Morris water maze: This test was performed with 12 to 21 weeks old mice. A circular tank (120 cm diameter) filled with opaque water of 20 to 22 °C was positioned in a room with distal cues visible to the swimming animal. Mice were repeatedly placed into the pool to locate a submerged escape platform (10 cm diameter) in an initial training phase. Spatial learning was assessed during 5 days across repeated trials, with 4 trials per day (“acquisition” phase). Starting positions varied from trial to trial according to a fixed scheme. After escape to the platform animals were allowed to spend 30 s on the platform. Animals that were not able to find the platform within 1 min were guided to and placed on the platform for 30 s. Reference memory was determined by preference of the platform area and average distance to platform-position when the platform was removed at day six of the experiment. Time during this retention-trial was limited to 1 min.

After acquisition and retention phase of the Water Maze experiment, a cued learning task was performed to test the ability of animals to swim straight and target-orientated to a proximal visual stimulus. Cued learning was assessed during four repeated trials. The submerged platform was cued with a dark-grey object (cone) that extends above the water surface. Starting position varied from trial to trial according to a fixed scheme. Animals that were able to locate and climb on the cued platform were allowed to spend 30 s on the platform. Finally, the position of the cued platform was moved and time to reach the platform was recorded.

### Antibodies

The TPC1 antibody was generated against the N-terminal Cys-peptide corresponding to amino acid region 774–790 of murine TPC1 (C-YQEEIQEWYEEHAREQE). Peptides were obtained from Intavis. The TPC2 antibody was generated by immunizing rabbits with the N-terminal Cys-peptide corresponding to amino acid region 705–726 of murine TPC2 (C-FRDILEEPKEEELMEKLHKHPH). Sera from immunized rabbits were peptide affinity purified employing the SulfoLink Immobilization Kit for Peptides (Thermo Scientific) according to the manufacturer´s protocol and an ÄKTAprime plus chromatography system (GE Healthcare Life Sciences). Antibody elution was performed by applying a linear pH gradient. The TPC1 antibody was first described in Arndt et al. (2014) and Castonguay et al. (2017); the TPC2 antibody first in Grimm et al. (2014).

Anti-alpha 1 sodium potassium ATPase antibody ab7671 was provided from abcam.

## Results

### Inactivation of the murine TPC1 gene

TPC1 was inactivated in the mouse genome using the Cre/loxP-recombination system and is schematically shown in Fig. 1a. Briefly, the knock out locus was generated by two rounds of Cre-dependent recombination. The first event deleted the neo-tk cassette and produced exon 3 flanked by loxP sites, the second one deleted exon 3 and generated a global TPC1 knock out. This strategy led to a frame shift in the TPC1 amino acid sequence and to a premature stop codon at amino acid residue 73 (Fig. 1b). In order to confirm the knock out and to get first insights into the TPC1 expression in the murine brain, we performed Western blot analysis for neocortex, hippocampus and cerebellum (Fig. 1c). TPC1 expression was detectable in all these brain regions of wild type mice, but not in TPC1^−/−^ mice. Since genetic deletion might cause upregulation of closely related genes (an example for L-type calcium channels is shown in (Xu et al. 2003), demonstrating an upregulation of the closely related L-type calcium channel Cav1.3 in Cav1.2-deficient murine hearts), we investigated expression of TPC2 in the murine brain of wild type and TPC1^−/−^ mice (Fig. 1c). TPC2 expression was unbalanced when comparing different brain regions and was high in the neocortex and hippocampus, but very low in the cerebellum. There was no compensatory upregulation of TPC2 in TPC1-deficient mice, indicating that expression of TPC2 is independent of TPC1.

## Behavioral phenotyping of TPC1^−/−^ mice

The deletion of the TPC1 gene did not result in any striking behavioral phenotype such as physical constraints, behavioral problems, aggressiveness or any other abnormal stereotypical behaviors. TPC1^−/−^ mice showed the same grooming and nest building behaviour as wild type mice. All animals used for further behavioral phenotyping experiments were routinely subjected to a test battery based on a modified SHIRPA test (Masuya et al. 2005). Animals were examined for the following parameters: freezing-behaviour, tremor, shortened, curled or damaged whiskers, streaming eyes, swollen or constricted eyelids, fur condition, piloerection, skin-colour, and trunk-curl and righting reflex. Throughout the survey period for these parameters, fear or aggression related behavior and vocalization was recorded. All these observations did not show any overt phenotype of TPC1^−/−^ animals.

There was no difference in body weight of 10-week-old mice (wild type: 25.12 ± 0.45 g; KO: 25.59 ± 0.49 g (MEAN ± SEM) and Supplementary Figure S1). As part of our basic characterization, we performed a wire hang test to analyze for an altered grip strength. Compared to wild type animals, TPC1^−/−^ mice demonstrated a significantly shorter latency to fall of the wires of the inverted lid [wild type: 54.03 ± 2.51 s; KO: 42.81 ± 3.58 s (mean ± SEM) (P = 0.020) and Supplementary Figure S2].

**Fig. 2:**
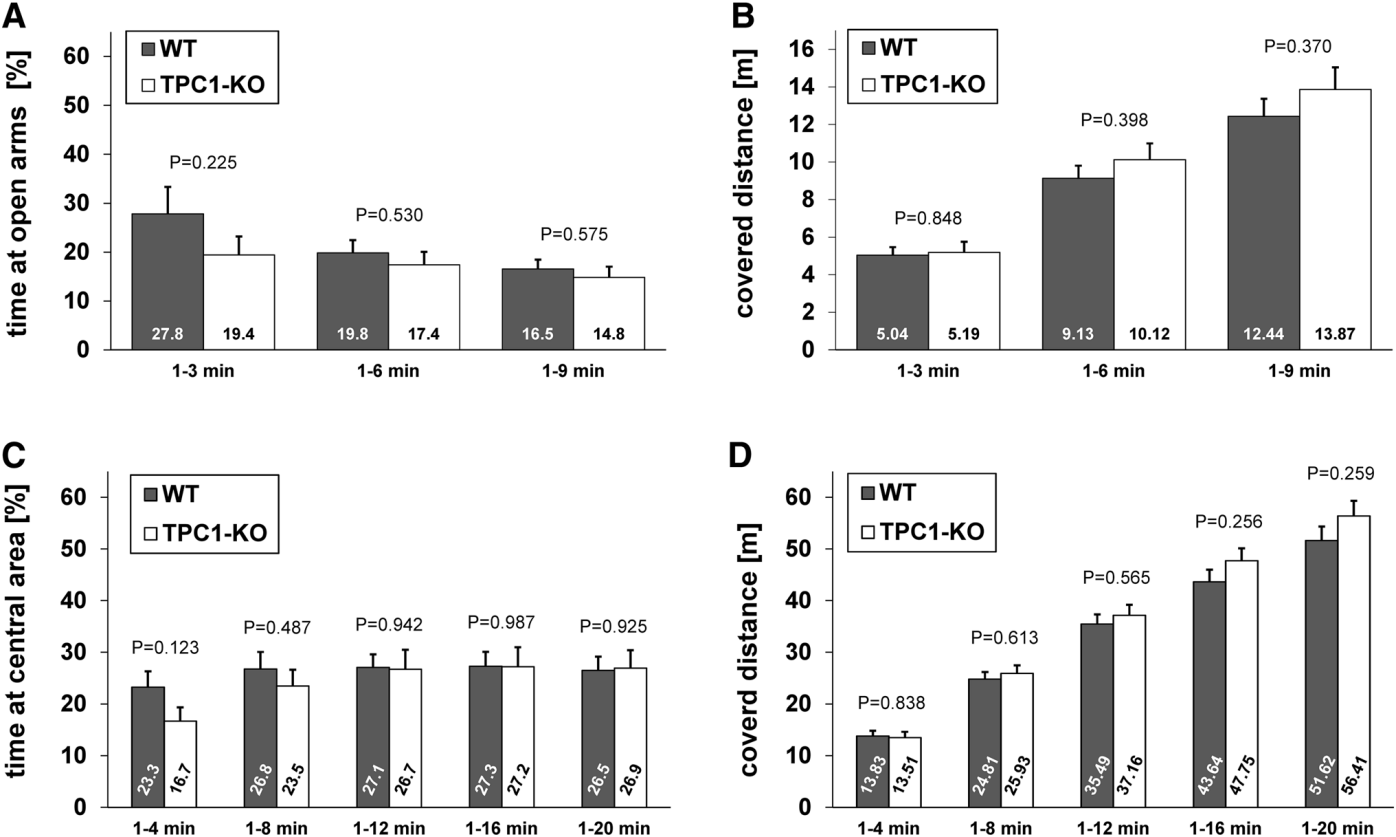
Elevated plus maze and open field test. Activity of 12 to 21 weeks old male wild type (N = 15) and TPC1^−/−^ mice (N = 17) was recorded over a period of 9 min during an elevated plus maze test. **a** Percentage of time spent at the open arms is presented for the first 3, 6 and 9 min. **b** Total covered distance for the same periods. **c** Spontaneous activity and exploratory behaviour of 10 weeks old male wild type (N = 15) and TPC1 knockout mice (N = 17) was recorded over a period of 20 min in the open field arena and percentage of time spent at central area is shown. **d** Total covered distance during the open field experiment. Statistical significance was assessed with two-tailed *t* test. Significance levels are indicated as P values

We studied anxiety-related behavior using the elevated plus maze. Mice were observed and separately evaluated for the first 3, 6 and 9 min by recording the time and measuring the covered distances on the four arms of the elevated plus maze. TPC1^−/−^ animals (N = 17) tested in the elevated plus maze, showed a tendency to spend shorter time periods at the open arms compared to their wild type littermates (N = 15) (Fig. 2a). However, this was not significantly different [percent of time at open arms (%): 1 to 3 min: WT 27.81 ± 5.54, KO 19.43 ± 3.75; 1 to 6 min: WT 19.84 ± 2.59, KO 17.40 ± 2.65 and 1 to 9 min: WT 16.55 ± 1.91, KO 14.83 ± 2.19 (mean ± SEM)]. There were also no significant differences, when measuring the covered distances at open arms and the number of open arm entries (Supplementary Figs. S3A and S3B). Furthermore, the total covered distances at the four arms were not significantly different between wild type and knockout mice (Fig. 2b) [total covered distances (m): 1 to 3 min: WT 5.04 ± 0.42, KO 5.19 ± 0.57; 1 to 6 min: WT 9.13 ± 0.67, KO 10.12 ± 0.87 and 1 to 9 min: WT 12.44 ± 0.92, KO 13.87 ± 1.17 (mean ± SEM)].

We further analyzed WT (N = 15) and TPC1^−/−^ mice (N = 17) in an open field arena, an additional test to study activity and anxiety-related behavior. Each mouse was observed for 20 min, but evaluation of the data was subdivided in time slots, each one four minutes longer than the previous one. Compared to wild type, TPC1^−/−^ mice spent a shorter time in the central area during the first minutes. However, this difference was not significant and further diminished during the ongoing test (Fig. 2c) [time at central area (%): 1 to 4 min: WT 23.28 ± 3.04, KO 16.69 ± 2.66; 1 to 8 min: WT 26.80 ± 3.28, KO 23.47 ± 3.17; 1 to 12 min: WT 27.09 ± 2.52, KO 26.73 ± 3.75; 1 to 16 min: WT 27.30 ± 2.81, KO 27.22 ± 3.78 and 1 to 20 min: WT 26.51 ± 2.65, KO 26.94 ± 3.46 (mean ± SEM)]. A comparable result was obtained, when measuring the covered distances at central area and the average distances to the center of the open field (Supplementary Figs. S4A and S4B). The total covered distance did not differ significantly for both genotypes (Fig. 2d) [total covered distances (m): 1 to 4 min: WT 13.83 ± 1.00, KO 13.51 ± 1.11; 1 to 8 min: WT 24.81 ± 1.39, KO 25.93 ± 1.57; 1 to 12 min: WT 35.49 ± 1.85, KO 37.16 ± 2.03; 1 to 16 min: WT 43.64 ± 2.36, KO 47.75 ± 2.37 and 1 to 20 min: WT 51.62 ± 2.74, KO 56.41 ± 2.92 (mean ± SEM)]. These results are in line with the observations from the elevated plus maze and indicate that TPC1^−/−^ mice do not differ in their explorative drive from wild type mice.

**Fig. 3:**
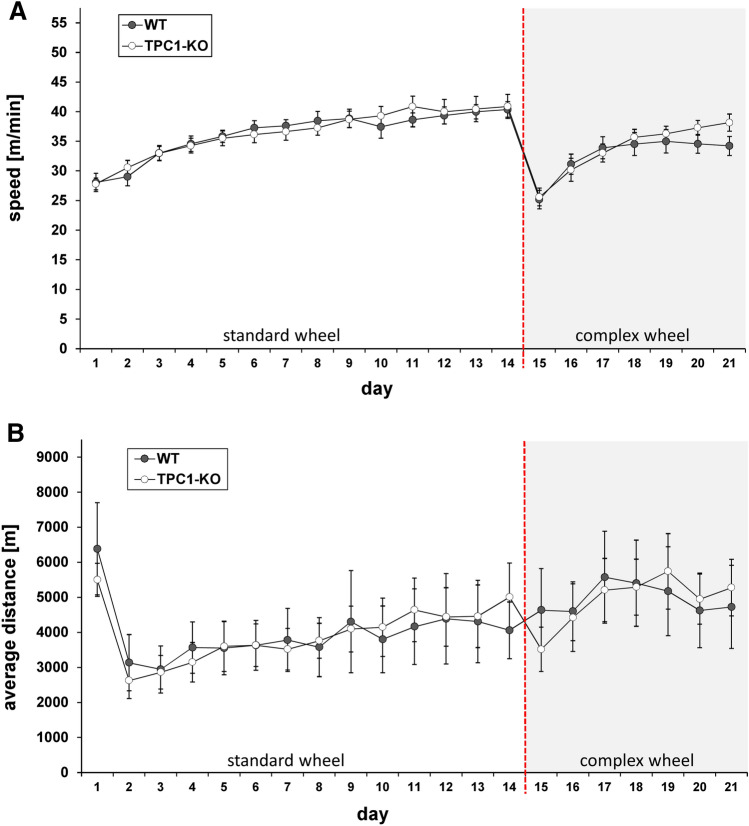
Voluntary wheel running. Single caged, adult male mice (12 to 28 weeks old) were allowed for voluntary wheel running during 21 days. At day 14 (indicated by the red dashed line) standard wheels (regular rung configuration) were replaced by “complex wheels”, meaning that particular rungs were removed to create an irregular rung configuration (Mandillo et al. 2014). Wheel running activity was recorded for wildtype (grey, N = 10) and TPC1^−/−^ mice (white, N = 10) and average running speed (**a**) and average distance (**b**) was calculated. Both parameters tested, showed no significantly different motor performances for WT and TPC1^−/−^ mice. Data are represented as mean ± SEM (Color figure online)

Next, we investigated spontaneous activity of TPC1^−/−^ mice in comparison with wild type mice in a voluntary running wheel setup. Wheel running activity was recorded first for 14 days for WT (N = 10) and TPC1^−/−^ mice (N = 10) and average running speed and average distance was calculated (Fig. 3a and b). During this period we did not observe any difference between the genotypes, indicating that TPC1^−/−^ show the same motivation for spontaneous activity than their WT littermates. At day 14 standard wheels (regular rung configuration) were replaced by “complex wheels”, meaning that particular rungs were removed to create an irregular rung configuration (Mandillo et al. 2014). Again, the same parameters, average speed and distances, were recorded until day 21 (Fig. 3a and b). Both parameters tested, showed no significantly different motor performances for WT and TPC1^−/−^ mice. Free running wheel activity was also used to test for an altered circadian rhythmicity. Since mice are nocturnal animals, they strongly prefer running during nighttime. However, evaluation of free running periods did not show differences between WT and TPC1^−/−^ mice with respect to running activity and time of day. We also tested for the number of runs per day, for the average time per run and for the average run-time per day. No parameters differed significantly between the genotypes (Supplementary Figure S5).

**Fig. 4:**
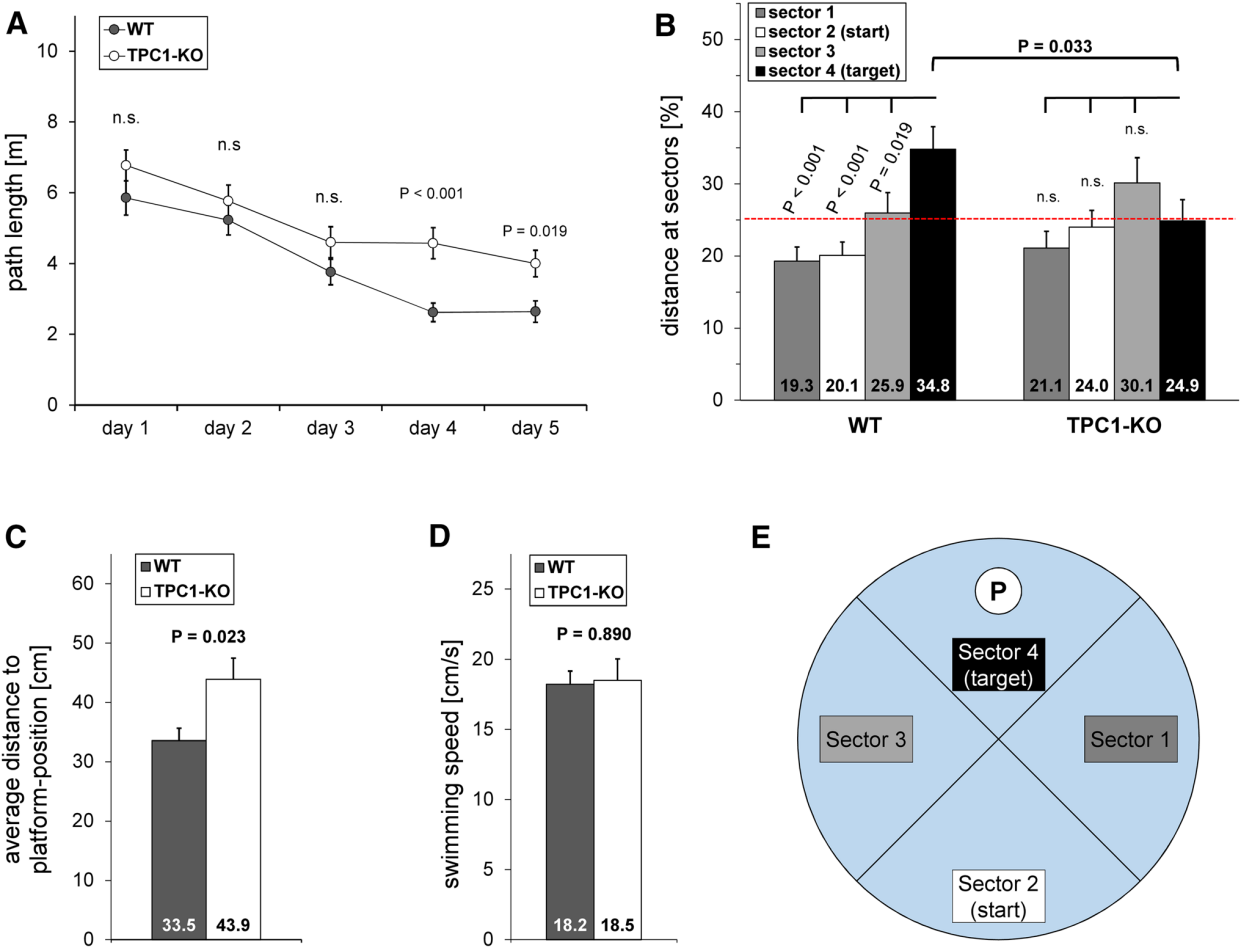
Morris water maze task. Figures present the results of the acquisition phase (**a**) and of the retention trial (**b**) to (**d**); **e** shows a scheme of the experimental setup of the Morris water maze (number and age of male mice: WT: N = 16 and TPC1^−/−^: N = 17; 12 to 21 weeks old). **a** Covered distance during day 1 to 5 of the acquisition phase. Data of the acquisition trials were averaged across four trials per day. Differences in path length were analyzed using two-way analysis of variance (ANOVA) with repeated measures and additional Holm–Sidak pairwise comparison procedure (WT vs. TPC1^−/−^ within each day). **b** Spatial memory was determined by preference of the platform (target) sector when the platform was removed at day 6 of the experiment (retention trial). Differences for sector occupancy (sector 1 – sector 4 within WT and TPC1^−/−^) were analyzed using one way analysis of variance (ANOVA). The Student–Newman–Keuls post-hoc test was used pairwise for multiple comparison. Differences in path length at sector 4 (target sector) between WT and TPC1^−/−^ mice was analyzed using two-tailed *t* test. **c** Average distance to platform position was measured as an independent parameter. Statistical significance was assessed with two-tailed *t* test. **d** Average swimming speed of mice during retention trial. Statistical significance was assessed with two-tailed *t* test. **e** Experimental setup of Morris water maze task indicating the arrangement of the four sectors. During retention animals were launched at quadrant two (start sector). Sector four (target sector) previously contained the submerged platform (“P” indicates platform position) during acquisition trials. Data are represented as mean + SEM, significance levels are indicated as P values; *n.s.* not significant (P > 0.05)

The Morris water maze test is a well-established spatial learning and memory task, which is known to depend on hippocampal function. During the acquisition phase TPC1^−/−^ mice demonstrated a comparable average path length to reach the platform at the first three days, but differed significantly at day 4 and 5 from their wild type littermates (Fig. 4a). The path length decreased stepwise from day 1 to day 5 from 5.85 ± 0.48, 5.23 ± 0.42, 3.76 ± 0.36, 2.62 ± 0.26 to 2.64 m ± 0.30 for wild type and from 6.77 ± 0.44, 5.77 ± 0.45, 4.60 ± 0.44, 4.57 ± 0.44 to 4.00 m ± 0.38 for knockout mice (mean ± SEM). Similar observations could be made for TPC1-deleted mice, when measuring the latency to escape that is the time to locate the position of the hidden platform (Supplementary Figure S6A).

During the retention trial at day 6, TPC1^−/−^ mice were not able to recall the platform-position as efficiently as wild type mice (Fig. 4b). In contrast to wild type mice, which demonstrated a significantly increased swimming distance in the target sector [distance at sectors (%): target sector 34.76 ± 3.13 vs. sector 1 19.26 ± 1.95, sector 2 20.05 ± 1.87 and sector 3 25.92 ± 2.86 (mean ± SEM)], TPC1^−/−^ mice did not prefer the target sector [distance at sectors (%): target sector 24.85 ± 2.94 vs. sector 1 21.09 ± 2.31, sector 2 23.97 ± 2.34 and sector 3 30.09 ± 3.52 (mean ± SEM)]. The same outcome was observed, when the time spent in the sectors was used to evaluate the retention trial (Supplementary Figure S6B). A very revealing parameter for the analysis of the Morris water maze is the measurement of the average distance of the mice to the platform position (Fig. 4c) (Gallagher et al. 1993). TPC1^−/−^ mice showed an average distance of 43.89 ± 3.57 cm to the platform position, whereas wild type mice were much closer (33.54 ± 2.08 cm) (mean ± SEM). Measurement of swimming speed as a control parameter did not show any differences between the genotypes [WT: 18.22 ± 0.94 cm/s, KO 18.48 ± 1.54 cm/s (mean ± SEM)], indicating that observed differences cannot be explained by varying swimming capabilities (Fig. 4d). Further control experiments measuring the speed and time to swim to a cued platform did also not show significant differences between the genotypes (Supplementary Figures S6C and S6D) [swimming speed to reach the cued platform: WT 15.62 ± 0.46 cm/s, KO 15.22 ± 0.53 cm/s; escape latency: WT 8.14 ± 0.80 s, KO 7.86 ± 0.58 s (mean ± SEM)]. Figure 4e presents a scheme of the experimental setup of the Morris water maze.

In summary, our behavioral studies indicate that TPC1^−/−^ mice do not differ from wild type mice with respect to exploratory drive, anxiety-like behavior, circadian rhythmicity and motor performance, but demonstrated a statistically significant impairment for spatial learning and memory.

## Discussion

In this study, we took advantage of our TPC1 knock out mice to achieve novel insights into higher order functions of this ion channel. Western blot experiments were performed in the murine brain to verify deletion of TPC1 and to exclude a possible upregulation of TPC2. Immunoblots indicated a broad expression of TPC1 throughout the murine brain including hippocampus and cerebellum. This rough estimation of course does not allow for a more detailed discussion of the precise roles of TPC1 in diverse brain regions. However, the data indicate at least that TPC1 might be involved in controlling of locomotor activity and learning and memory. Thus, we aimed to achieve a basic behavioural characterization of our TPC1 knock out mice.

The broad expression of TPC1 in the murine brain suggests that its genetic deletion might cause rather diverse behavioral phenotypes. We investigated TPC1^−/−^ mice for an altered exploratory drive, anxiety related behavior and for learning and memory. Results from open field and elevated plus maze are in line, since both tests suggest that TPC1^−/−^ mice show an exploratory drive comparable to wild type mice. Possibly TPC1^−/−^ mice were more receptive towards aversive stimuli during the first minutes in a new, unfamiliar environment. However, this observation was a subjective assessment and was statistically not proven. Locomotor behavior and locomotor coordination as well as circadian rhythmicity did not differ between wild type and TPC1^−/−^ mice.

The wire hanging grip strength test indicated that TPC1^−/−^ mice demonstrated a significantly shorter latency to fall off the inverted wire mesh. Since our previous study (Castonguay et al. 2017) did not indicate a significant expression of TPC1 in skeletal muscle in Western blots, the reason for the grip strength phenotype is hard to explain and we assume that this result was not caused by an altered muscle performance. Motor neuron dysfunction may be an explanation for such a phenomenon as well as an altered anaerobic metabolism that occurs during the wire hanging test. However, no experimental data support one of these issues.

In contrast to above mentioned studies investigating exploratory drive and anxiety, the results from the Morris water maze reached significant levels. At day four and five of the acquisition phase, TPC1^−/−^ mice significantly required longer times and covered longer distances to find the position of the hidden platform than wild type mice. Evaluation of the parameters time and distance at target sector during the retention phase indicated an impairment of place learning and spatial memory for TPC1^−/−^ mice. In control experiments, comparable cued learning performances of WT and TPC1^−/−^ mice demonstrated intact abilities, such as eyesight and adequate motor skills as well as normal motivation and an effective strategy to escape from the water.

Impaired learning as seen in the Morris water maze might be caused by alterations in glutamatergic signal transduction in the hippocampus. It was shown that application of glutamate to hippocampal cell culture evoked cytosolic Ca^2+^ signals that were independent from external Ca^2+^, but depended on acidic pH and integrity of endolysosomes and it was found to increase neuronal NAADP levels (Pandey et al. 2009). Additionally, the sensitivity for glutamate correlated with Ca^2+^ levels in endolysosomes. Refilling of acidic compartments by addition of extracellular Ca^2+^ recovered glutamate-induced Ca^2+^ release (Pandey et al. 2009). This is of particular interest since metabotropic glutamate receptors have been shown to be linked to IP_3_ synthesis (Fagni et al. 2000). Taken together, these data point to an NAADP-induced Ca^2+^ release mechanism in endolysosomes as a prerequisite for an IP_3_-induced Ca^2+^ release from the ER for metabotropic receptor mediated Ca^2+^ signaling (Galione 2015). However, in a recent study, this mechanism was queried and it was suggested that SK-type K^+^ channels play a central role for facilitating LTP (Foster et al. 2018). According to their results, activation of metabotropic glutamate receptor mGluR1 causes NAADP synthesis, which in turn increases Ca^2+^ levels by TPC-mediated Ca^2+^ release from acidic stores and subsequent amplification by ER Ca^2+^ release. Selective inhibition of ER Ca^2+^ release mechanisms demonstrates that amplification of Ca^2+^ signals depends on ryanodine receptors, but does not require IP3 receptors. The elevated Ca^2+^ levels transiently inhibit SK channels, presumably by activation of protein phosphatase 2A. Consequently local membrane hyperpolarization is prevented and a higher Ca^2+^ influx via GluN receptors occurs and facilitates induction of LTP (Foster et al. 2018). Thus, deletion of TPC1 may interfere with this Ca^2+^-dependent processes and might be the reason for modulation of synaptic transmission, affecting learning and memory performance of TPC^−/−^ mice.

One should keep in mind that TPCs play a central role for intracellular trafficking and thereby for regulation of surface expression of membrane proteins via endocytosis and recycling processes. In this context, the regulation of surface accessible AMPA receptors may be of critical importance since this class of ionotropic glutamate receptors also affects excitatory synaptic transmission and synaptic plasticity, which forms the molecular correlate for learning and memory (Moretto and Passafaro 2018; Park 2018; Parkinson and Hanley 2018). It is obvious to hypothesize that deletion of TPCs disturbs AMPA receptor trafficking at various levels. Relevant mechanisms may include exocytosis from recycling endosomes, lateral diffusion to synaptic sites, and surface retrieval by endocytosis and lysosomal degradation.

The global deletion of TPC1 in our mouse model might also cause distinct neurodevelopmental deficits. Possibly, development of hippocampus might be disturbed by TPC1-deficiency and could be the cause for the observed learning interferences in the Morris water maze. Therefore, we suggest future studies using conditional neuronal knockouts, such as Cre/loxP mediated forebrain specific mouse models (Mallmann et al. 2013) to figure out the precise neuronal pathways that underlie the observed phenotype in TPC1-deficient mice, i.e. the impaired learning and memory.

## Electronic supplementary material

Below is the link to the electronic supplementary material.

Supplementary material 1 (DOCX 612 kb)
