# Supplementary material for: Genetic Inactivation of Two-Pore Channel 1 Impairs Spatial Learning and Memory
Source: Behav Genet. 2020 Sep 5;50(6):401–10. doi: 10.1007/s10519-020-10011-1 (PMC7581579; doi:10.1007/s10519-020-10011-1)
Supplement: Supplementary file 1 — Supplementary material 1 (DOCX 612 kb) [file 10519_2020_10011_MOESM1_ESM.docx]

**Genetic inactivation of two-pore channel 1 impairs spatial learning and memory**

Robert Theodor Mallmann^1^ & Norbert Klugbauer^1^

^1^Institut für Experimentelle und Klinische Pharmakologie und Toxikologie, Medizinische Fakultät, Albert-Ludwigs-Universität, Freiburg, Germany;

Supplementary figures S1 to S6

**
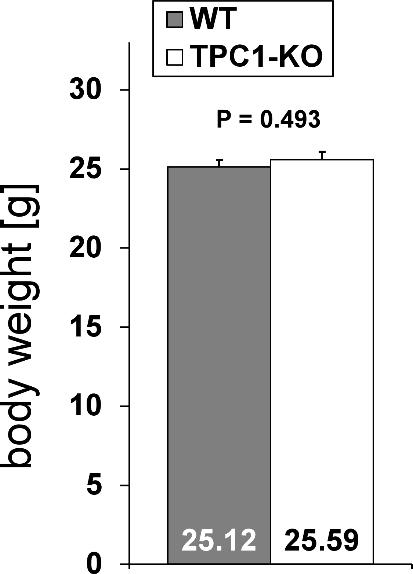
**

**Supplementary Figure S1: Body weight** **of ten weeks old, male wild type (N=21) and TPC1^-/-^ (N=20) mice.** Statistical significance was assessed with two-tailed t-test. Significance level is indicated as P value.

**
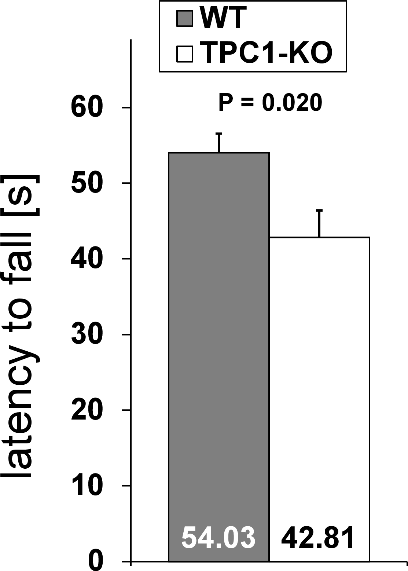
**

**Supplementary Figure S2: Wire hanging grip strength test.** Ten weeks old male wild type (N=15) and TPC1^-/-^ (N=16) mice were placed in the middle of a wire cage lid. Latency of mice to fall off the inverted wire mesh was timed. Animals were tested in two consecutive trials with 1 h interval between trials. A 60 seconds cut-off time was used for each mouse tested. Statistical significance was assessed with two-tailed t-test. Significance level is indicated as P value.

**
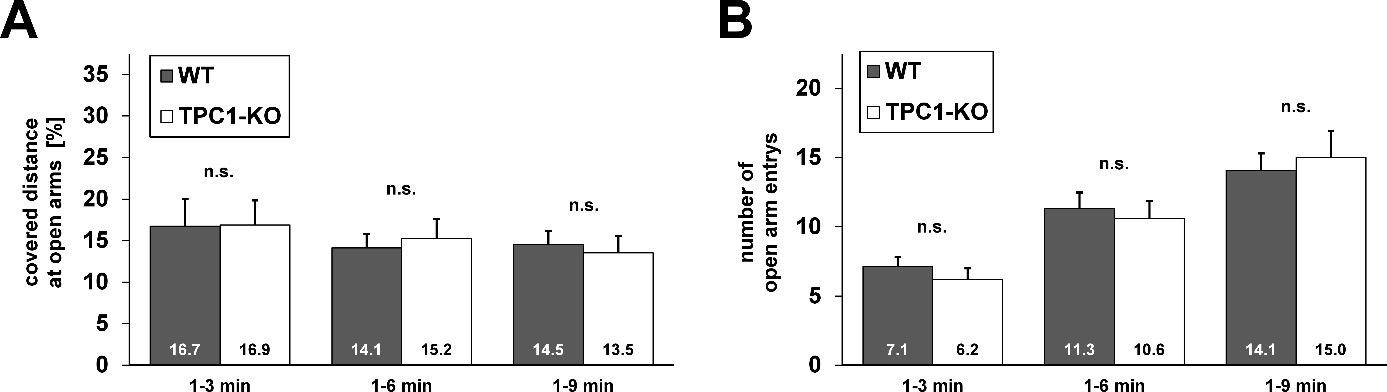
**

**Supplementary Figure S3:** **Additional data for the elevated plus maze of male wild type (N=15) and TPC1^-/-^ mice (N=17).** (A) Percentage of covered distance at open arms is presented for the first 3, 6 and 9 minutes. (B) Number of open arm entries for the same periods. Statistical significance was assessed with two-tailed t-test. Data are represented as MEAN + SEM, significance levels are indicated as P values; n.s., not significant (P > 0.05).

**
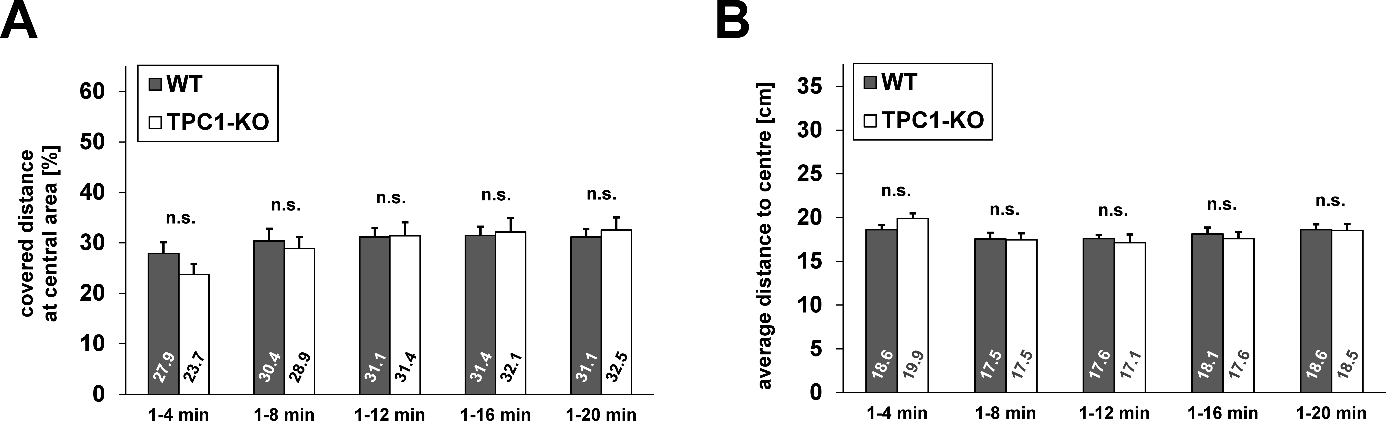
**

**Supplementary Figure S4:** **Additional data for the open field arena of male wild type (N=15) and TPC1^-/-^ mice (N=17).** (A) Percentage of covered distance at the central area of the open field arena. (B) Average distance to open field arena-centre. Statistical significance was assessed with two-tailed t-test. Data are represented as MEAN + SEM, significance levels are indicated as P values; n.s., not significant (P > 0.05).

**
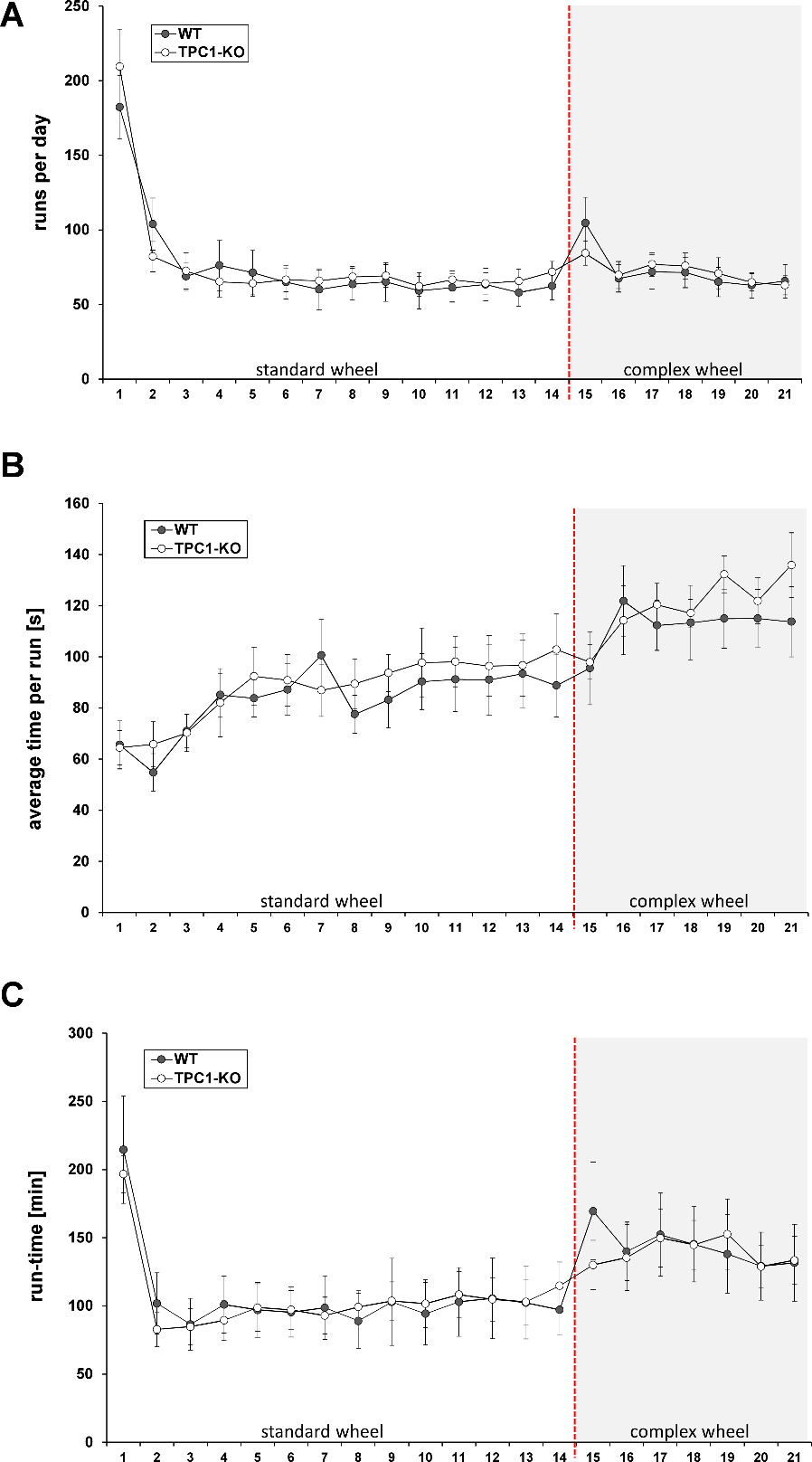
**

**Supplementary Figure S5: Additional data for the voluntary wheel running.** Single caged, adult male mice (12 to 28 weeks old) were allowed for voluntary wheel running during 21 days (wildtype (grey, N=10) and TPC1^-/-^mice (white, N=10)). At day 14 (indicated by the red dashed line) standard wheels were replaced by “complex wheels”, meaning that particular rungs were removed to create an irregular rung configuration. The following parameters were recorded: (A) The number of runs per day, (B) the average time per run and (C) the average run-time. All parameters tested, showed no significantly different motor performances for WT and TPC1^-/-^ mice. Data are represented as MEAN ± SEM.

**
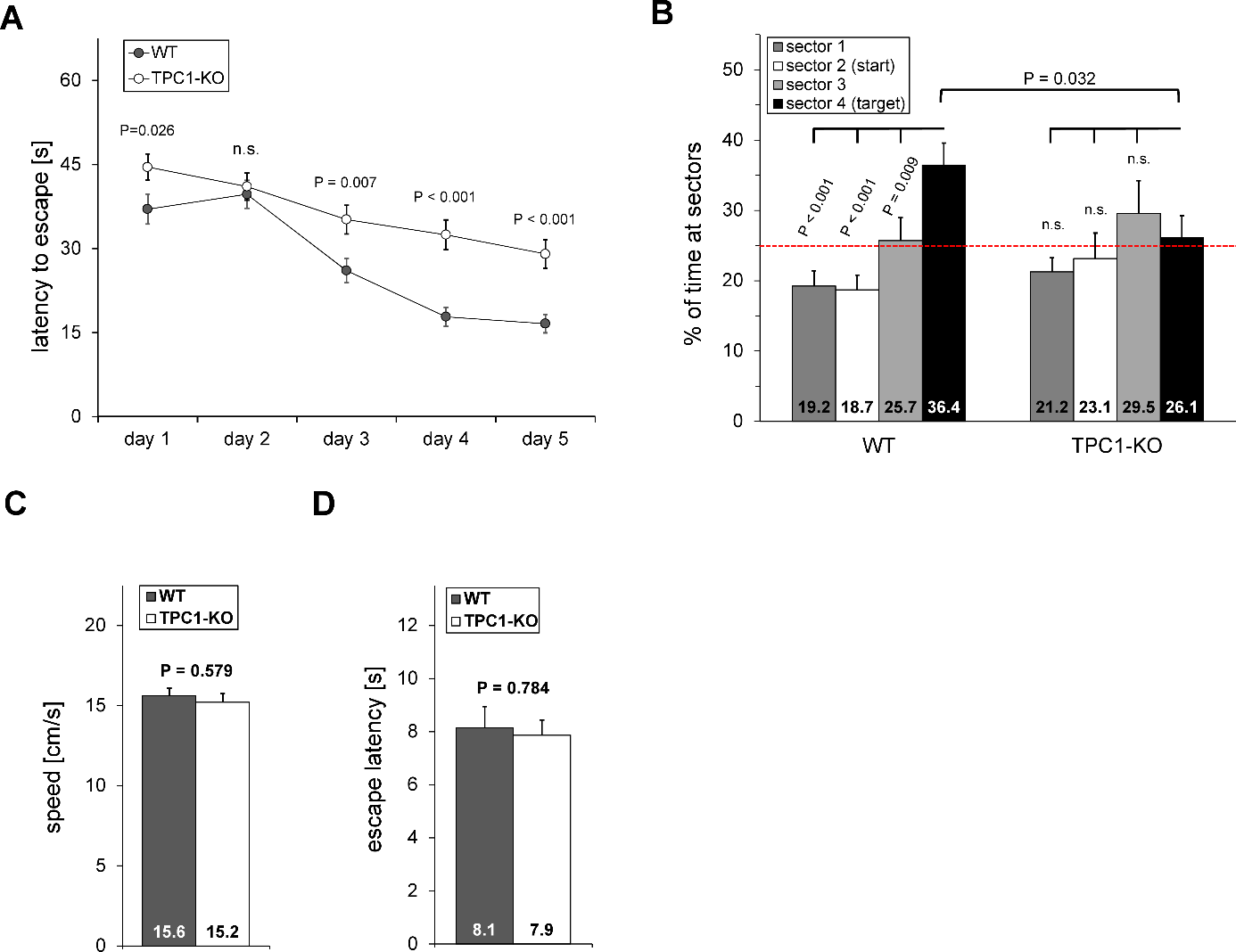
**

**Supplementary Figure S6: Additional parameters tested during Morris water maze acquisition (A) and retention phase (B) of wild type (N=16) and TPC1^-/-^ (N=17) mice. (C) & (D) displays the performance of the mice tested during cued learning.** (A) Latency to escape during day 1 to 5 of the acquisition phase. Data of the acquisition trials were averaged across four trials per day. Differences in escape latencies were analyzed using two-way analysis of variance (ANOVA) with repeated measures and additional Holm-Sidak pairwise comparison procedure (WT vs. TPC1-KO within day). (B) Spatial memory was determined by preference of the platform (target) sector when the platform was removed at day 6 of the experiment (retention trial). Differences for sector occupancy (sector 1 – sector 4 within WT and TPC1-KO) was analyzed using one way analysis of variance (ANOVA). The Student-Newman-Keuls post-hoc test was used pairwise for multiple comparison. Differences in percentages of time at sector 4 (target sector) between WT and TPC1-KO mice was analyzed using two tailed t-test. (C) Average swimming speed and (D) latency to escape during cued learning. This experiment is a control procedure routinely used during the Morris water maze task. Statistical significance was assessed with two-tailed t-test. Data are represented as MEAN + SEM, significance levels are indicated as P values; n.s., not significant (P > 0.05).
